# Disruption of the interaction between mutationally activated Gα_q_ and Gβγ attenuates aberrant signaling

**DOI:** 10.1016/j.jbc.2023.102880

**Published:** 2023-01-07

**Authors:** Jenna L. Aumiller, Philip B. Wedegaertner

**Affiliations:** Department of Biochemistry and Molecular Biology, Sidney Kimmel Medical College, Thomas Jefferson University, Philadelphia, Pennsylvania, USA

**Keywords:** Heterotrimeric G protein, cell signaling, GTPase, oncogene, uveal melanoma, BSA, bovine serum albumin, CA, constitutively active, FBS, fetal bovine serum, GPCR, G protein–coupled receptor, IP, inositol phosphate, MAPK, mitogen-activated protein kinase, pERK, phospho-ERK, PLC-β, phospholipase C–β, SRE, serum response element, TBS, tris-buffered saline, TEAD, TEA domain, UM, uveal melanoma, YAP, Yes-associated protein

## Abstract

Heterotrimeric G protein stimulation *via* G protein–coupled receptors promotes downstream proliferative signaling. Mutations can occur in Gα proteins which prevent GTP hydrolysis; this allows the G proteins to signal independently of G protein–coupled receptors and can result in various cancers, such as uveal melanoma (UM). Most UM cases harbor Q209L, Q209P, or R183C mutations in Gα_q/11_ proteins, rendering the proteins constitutively active (CA). Although it is generally thought that active, GTP-bound Gα subunits are dissociated from and signal independently of Gβγ, accumulating evidence indicates that some CA Gα mutants, such as Gα_q/11_, retain binding to Gβγ, and this interaction is necessary for signaling. Here, we demonstrate that disrupting the interaction between Gβγ and Gα_q_ is sufficient to inhibit aberrant signaling driven by CA Gα_q_. Introduction of the I25A point mutation in the N-terminal α helical domain of CA Gα_q_ to inhibit Gβγ binding, overexpression of the G protein Gα_o_ to sequester Gβγ, and siRNA depletion of Gβ subunits inhibited or abolished CA Gα_q_ signaling to the MAPK and YAP pathways. Moreover, in HEK 293 cells and in UM cell lines, we show that Gα_q_-Q209P and Gα_q_-R183C are more sensitive to the loss of Gβγ interaction than Gα_q_-Q209L. Our study challenges the idea that CA Gα_q/11_ signals independently of Gβγ and demonstrates differential sensitivity between the Gα_q_-Q209L, Gα_q_-Q209P, and Gα_q_-R183C mutants.

Heterotrimeric G proteins (Gαβγ) are canonically activated through G protein–coupled receptors (GPCRs), which allows for GDP release from the Gα subunit and further GTP binding. It is generally accepted that GTP binding on the Gα subunit promotes an active conformation and allows for the dissociation from Gβγ. Active GTP-bound Gα proteins can further bind and stimulate downstream effectors (1, 2). Hydrolysis of GTP on the Gα subunit promotes reassociation of the inactive Gαβγ heterotrimer and completes the cycle. Several conserved amino acids, found in all Gα subunits, are critical for the intrinsic GTP hydrolysis activity, and mutations of these residues prevent GTP hydrolysis and “lock” the Gα protein in a constitutively active (CA), GTP-bound state (3, 4). Aberrant CA mutations in Gα proteins have been identified in various human tumors and diseases. For example, Gα_s_ CA mutations have been identified in a subset of pancreatic tumors (5, 6). Mutations in Gα_q_ and Gα_11_ have also been described at residues Q209 and R183 in the majority of patients with uveal melanoma (UM). Q209L or Q209P mutations in Gα_q_ or Gα_11_ occur in 90% of UM patients, and mutations at R183 occur in about 5% of patients (7, 8, 9, 10). Approximately, 50% of patients with UM develop distant metastatic disease to the liver; there are currently no effective therapies for metastatic UM. Upon metastatic diagnosis, the average survival is only 2 to 8 months, indicating the urgent importance of effective therapies for metastatic UM (11, 12).

The constitutive activity of Gα_q/11_ in UM, driven by the activating Q209L/P and R183 mutations, promotes the stimulation of two major signaling pathways: 1) the mitogen-activated protein kinase (MAPK) pathway and 2) the Yes-associated protein and Transcriptional coactivator with PDZ-binding motif (YAP/TAZ) pathway. The MAPK cascade is stimulated by the direct binding of CA Gα_q/11_ to phospholipase C–β (PLC-β), which hydrolyzes phosphatidylinositol 4,5-bisphosphate into diacylglycerol and inositol 1,4,5-trisphosphate. Diacylglycerol further activates PKC, which phosphorylates and activates RasGRP3. The further activation of Ras promotes the stimulation of the MAPK cascade and results in the phosphorylation and activation of ERK. Phospho-ERK (pERK) dimerizes and translocates into the nucleus to bind transcription factors and promote the transcription of proliferative genes (13). Mutationally active Gα_q/11_ also stimulates the YAP/TAZ pathway through direct binding and activation of the RhoGEF Trio. In an inactive state, YAP is phosphorylated and remains cytoplasmic through mediators of the Hippo pathway. Activation of Trio leads to the stimulation of focal adhesion kinase, which inhibits mediators of the Hippo pathway and ultimately leads to the dephosphorylation and nuclear translocation of YAP. YAP binds to the TEA domain (TEAD) transcription factor in the nucleus to promote the transcription of genes involved in proliferation and cell survival (14, 15). To date, inhibitors for downstream targets of CA Gα_q/11_ have been proven to be generally unsuccessful in disrupting the progression of metastatic UM (16, 17, 18).

Because Gα_q/11_ stimulates multiple pathways, directly targeting CA Gα_q/11_ may be a more promising therapeutic strategy for metastatic UM patients. There are currently no FDA-approved drugs that target Gα_q/11_; however, the compounds YM-254890 (YM) and FR900359 (FR) have been shown to inhibit both WT and CA Gα_q/11_ and have shown promising results in cancer cell models (19, 20, 21). YM and FR have similar structures and are thought to prevent the release of GDP from Gα_q/11_ and ultimately inhibit GDP to GTP exchange (21, 22, 23). However, both YM and FR to date have had varying effects on UM tumor arrest and regression in preclinical UM mouse models (24, 25). Therefore, understanding how CA Gα_q/11_ is regulated in cells and further exploring other methods of inhibiting oncogenic Gα_q/11_ may provide therapeutic benefits for metastatic UM patients.

In an inactive, GDP-bound state, Gα proteins form heterotrimers with Gβγ. This interaction with Gβγ aids in plasma membrane localization to neighboring GPCRs and helps stabilize the complex for GTP exchange (26, 27, 28, 29, 30, 31). Crystal structures indicate that the inactive, GDP-bound Gα subunit has two main points of contact with Gβγ: 1) a central switch region, which undergoes a conformational change upon GTP binding and 2) an N-terminal α helical domain (32, 33, 34). It is generally accepted in the field that during nucleotide exchange, the Gα subunit can dissociate from the Gβγ subunit; therefore, it would be reasonable to assume that the CA Gα_q/11_ mutants commonly found in UM would have minimal interaction with the Gβγ. However, work in our lab and others suggest that the CA Gα_q_-Q209L mutant retains a functionally important interaction with Gβγ *via* the N-terminal α helical domain (32, 33, 34). Previous work in our lab indicates that a single point mutation (I25A) in the N-terminal α helix of WT Gα_q_ is sufficient to disrupt binding to Gβγ, and this disruption of binding further prevents GPCR-dependent signaling of WT Gα_q_ (35). The I25A N-terminal Gβγ-binding mutation was also sufficient to disrupt overactive inositol phosphate (IP) production for the CA Gα_q_-R183C mutant. Understanding the role of Gβγ in the oncogenic signaling activity of CA Gα_q_ could address the gaps in knowledge of the cellular regulation of CA Gα_q_ and further offer Gβγ as a potential target for disrupting aberrant signaling in UM.

Here, we tested the hypothesis that disrupting the interaction between Gβγ and the CA Gα_q_ mutants commonly found in UM patients—Q209L (QL), Q209P (QP), and R183C (RC)—is sufficient to inhibit oncogenic signaling of these mutationally active Gα_q_. We provide evidence that disrupting the interaction between CA Gα_q_ and Gβγ significantly inhibits Gα_q_-mediated activation of the MAPK and YAP/TAZ pathways. Surprisingly, we show differential sensitivity between Gα_q_-QL and the Gα_q_-QP/RC mutants in that oncogenic signaling by Gα_q_-QP and Gα_q_-RC is more sensitive to the disruption of Gβγ binding than Gα_q_-QL.

## Results

### N-terminal Gβγ-binding mutation I25A inhibits oncogenic signaling by CA Gα_q_ mutants

**Figure 1:**
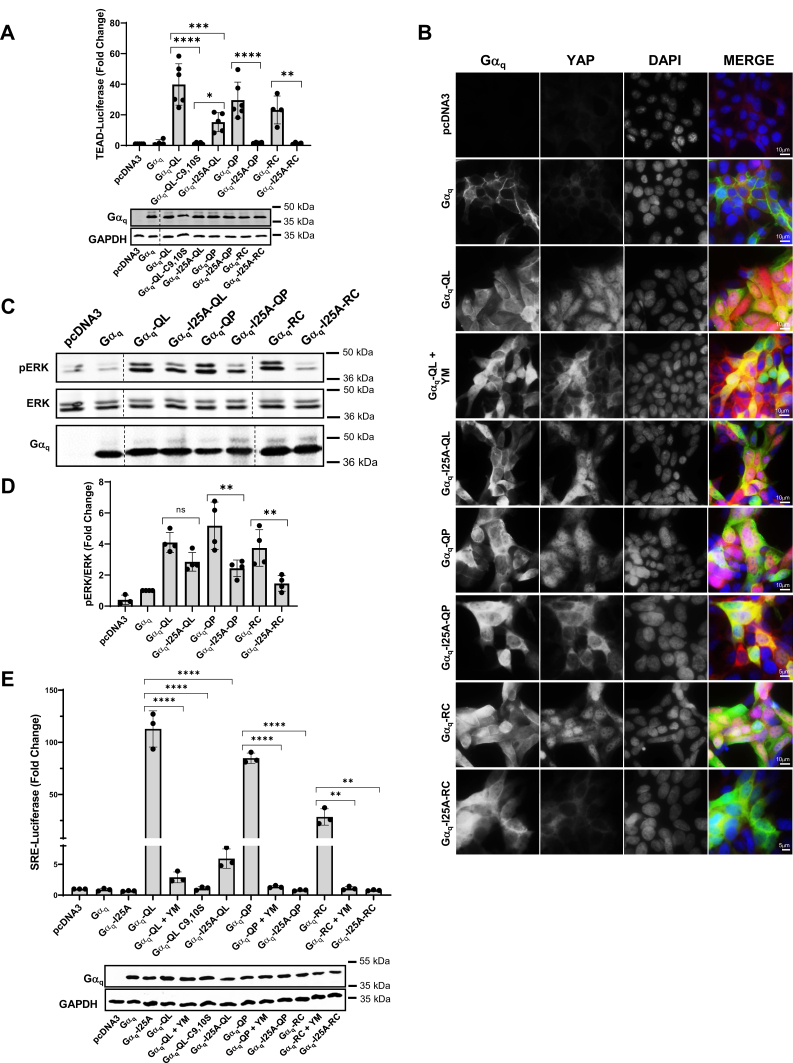
**Oncogenic signaling by constitutively active Gα**_**q**_**is inhibited by I25A N-terminal Gβγ-binding mutation.***A*, HEK 293 Gα_q/11_ KO cells were transfected with pcDNA3, WT Gα_q_, Gα_q_-QL, Gα_q_-QL-C9,10S, Gα_q_-I25A-QL, Gα_q_-QP, Gα_q_-I25A-QP, Gα_q_-RC, or Gα_q_-I25A-RC, along with 8x-GTIIC Luciferase and Renilla control plasmids. Cell lysates were prepared, and luciferase assays were performed and quantified. Graph indicates fold change over pcDNA3. Results are shown as mean ± SD. Statistical significance is indicated (n = 6 for Gα_q_-QL, Gα_q_-QP; n = 5 for pcDNA3, Gα_q_-QL-C9,10S, Gα_q_-I25A-QL; n = 4 for WT Gα_q_, Gα_q_-I25A-QP, Gα_q_-RC, Gα_q_-I25A-RC, ∗*p* < 0.05; ∗∗*p* < 0.01; ∗∗∗*p* < 0.005; ∗∗∗∗*p* < 0.0001, two-way ANOVA, Šidák's multiple comparison’s test). Lysates were also immunoblotted to detect relative expression levels of the various Gα_q_ mutants along with GAPDH controls, and a representative immunoblot is shown. *B*, HEK 293 Gα_q/11_ KO cells transfected with pcDNA3, WT Gα_q_, Gα_q_-QL, Gα_q_-I25A-QL, Gα_q_-QP, Gα_q_-I25A-QP, Gα_q_-RC, or Gα_q_-I25A-RC. Gα_q_-QL + YM indicates that cells were transfected with Gα_q_-QL and treated with 1 μM YM for 16 h. Coverslips were processed for immunofluorescence microscopy to detect Gα_q_, YAP, and nuclei (DAPI) (n = 4) as described under Experimental procedures, and representative images are shown. *C* and *D*, HEK 293 Gα_q/11_ KO cells were transfected with pcDNA3, WT Gα_q_, Gα_q_-QL, Gα_q_-I25A-QL, Gα_q_-QP, Gα_q_-I25A-QP, Gα_q_-RC, or Gα_q_-I25A-RC. *C*, cell lysates were prepared and immunoblotted for pERK, ERK, and Gα_q_. *D*, pERK/ERK signal intensities were quantified. Graph indicates fold change of pERK/ERK levels over WT Gα_q_. Results are shown as mean ± SD. Statistical significance is indicated. (n = 4; n = 3 for pcDNA3, ∗∗*p* < 0.01, two-way ANOVA, Šidák's multiple comparison’s test). *E*, HEK 293 Gα_q/11_ KO cells were transfected with pcDNA3, WT Gα_q_, Gα_q_-I25A, Gα_q_-QL, Gα_q_-QL-C9,10S, Gα_q_-I25A-QL, Gα_q_-QP, Gα_q_-I25A-QP, Gα_q_-RC, or Gα_q_-I25A-RC, along with SRE Luciferase and Renilla control plasmids. Gα_q_-QL, Gα_q_-QP, and Gα_q_-RC conditions were treated with 1 μM YM for 16 h as indicated. Lysates were immunoblotted to detect Gα_q_ and GAPDH protein levels. Cell lysates were prepared and luciferase assays were performed and quantified. Results are shown as mean ± SD. Graph indicates fold change over pcDNA3. Statistical significance is indicated (n = 3; ∗∗*p* < 0.01; ∗∗∗∗*p* < 0.0001, two-way ANOVA, Šidák's multiple comparison’s test). pERK, phospho-ERK; YAP, Yes-associated protein.

Previous work has shown that the activation of downstream effectors of Gα_q_, such as PLC-β, requires the binding of Gα_q_ to Gβγ (35). Furthermore, recent studies have indicated that CA Gα_q_-Q209L proteins retain binding to Gβγ at the N-terminal α-helical region (36). It has been established that the I25A point mutation within the N-terminal α-helical domain of Gα_q_ is sufficient to disrupt the interaction between Gα_q_ and Gβγ and consequently inhibit the GPCR-dependent signaling of WT Gα_q_ (35). Thus, we hypothesized that introducing the I25A mutation to disrupt binding to Gβγ would inhibit oncogenic signaling through the CA Gα_q_-Q209L (QL), Q209P (QP), and R183C (RC) mutants. To monitor the activity of the YAP/TAZ pathway, we transfected the CA mutants, with and without the I25A mutation, into HEK 293 Gα_q/11_ CRISPR-Cas9 KO cells and monitored YAP activity using the TEAD luciferase reporter assay, which is stimulated in response to YAP activation and nuclear translocation. Gα_q_-QL, Gα_q_-QP, and Gα_q_-RC all showed strong activation in the assay. Cells expressing the pcDNA3 vector and WT Gα_q_ were used as negative controls and indicated no significant change in TEAD luciferase reporter activity (Fig. 1*A*). The Gα_q_-QL-C9,10S palmitoylation-deficient mutant was used as a negative control for Gα_q_-QL. The C9,10S mutation in WT Gα_q_ has been previously established to prevent plasma membrane localization and consequently prevent GPCR-dependent activation (37); likewise, we now show that Gα_q_-QL-C9,10S is unable to stimulate TEAD-dependent luciferase activity. Interestingly, Gα_q_-I25A-QL displayed a decreased ability compared to Gα_q_-QL to activate TEAD luciferase activity, yet retained significantly higher activity than the Gα_q_-QL-C9,10S control. In contrast, the introduction of the I25A mutation abolished the ability of Gα_q_-QP and Gα_q_-RC to stimulate TEAD luciferase. These data demonstrate that the introduction of the I25A mutation inhibits oncogenic signaling to the YAP pathway by the CA Gα_q_-QL/P and Gα_q_-RC mutants. These results also suggest potential differential sensitivity between the CA Gα_q_-QL and Gα_q_-QP/RC mutants.

To further monitor YAP activity and the differential sensitivity between the CA Gα_q_-I25A mutants, we used immunofluorescence microscopy to examine the cellular localization of YAP (Fig. 1*B*). It has been previously established that activation of the Rho-dependent YAP pathway results in stabilization and dephosphorylation of YAP and its subsequent translocation into the nucleus (38, 39). The YAP pathway was not stimulated in cells expressing the pcDNA3 vector and WT Gα_q_, as shown by the low expression and cytoplasmic localization of YAP. Expression of Gα_q_-QL indicated strong nuclear localization of YAP, and treatment with the Gα_q/11_ inhibitor YM-254890 (YM) as a control resulted in the loss of nuclear YAP and its accumulation in the cytoplasm. Gα_q_-I25A-QL also promoted nuclear localization of YAP, consistent with its retention of signaling in the TEAD-luciferase assay (Fig. 1*A*). Cells expressing CA Gα_q_-QP and Gα_q_-RC had robust nuclear YAP localization, indicating stimulation of this pathway. However, Gα_q_-I25A-QP and Gα_q_-I25A-RC failed to stimulate the nuclear localization of YAP, as indicated by the cytoplasmic localization of YAP. Similar to the TEAD-luciferase reporter assay results, this data suggests that Gα_q_-I25A-QL is more resistant to disruption of signaling than the Gα_q_-I25A-QP and Gα_q_-I25A-RC mutants. In immunofluorescence microscopy studies, we also examined the subcellular localization of the CA Gα_q_ mutants with and without the I25A mutation (Fig. S1). Previous work has demonstrated that the interaction with Gβγ is crucial for palmitoylation and further membrane localization of Gα_q_ (27). Gα_q_-QL and Gα_q_-RC were localized to the plasma membrane in transfected cells. The introduction of the I25A mutation does not significantly impact the plasma membrane localization of Gα_q_-QL but results in partial loss of plasma membrane localization of Gα_q_-RC (Fig. S1). Interestingly, Gα_q_-QP had only partial plasma membrane association, and introduction of the I25A mutation resulted in an almost complete loss of plasma membrane localization (Fig. S1). Thus, decreased plasma membrane localization of Gα_q_-RC and Gα_q_-QP when interaction with Gβγ is disrupted through introduction of the I25A mutation provides at least a partial mechanistic explanation for their loss of signaling. These differences in cellular localization of the Gα_q_-I25A-QL, Gα_q_-I25A-QP, and Gα_q_-I25A-RC (Fig. S1) likely contribute to the differential sensitivity in aberrant cell signaling (Fig. 1).

Along with stimulation of the YAP pathway, CA Gα_q/11_ also canonically stimulates PLC-β and further activates the MAPK cascade. To monitor the effects of the I25A mutants on the MAPK pathway, we quantified pERK levels in HEK 293 Gα_q/11_ KO cells transfected with the CA Gα_q_ constructs and the I25A mutants (Fig. 1, *C* and *D*). Expression of CA Gα_q_-QL resulted in pERK stimulation >4-fold compared to cells transfected with pcDNA3 or WT Gα_q_. The Gα_q_-I25A-QL mutant also activated pERK at a slightly decreased level compared to Gα_q_-QL, although the difference was not statistically significant. Interestingly, the Gα_q_-QP and Gα_q_-RC mutants stimulated pERK, but pERK activity was significantly decreased in cells expressing the Gα_q_-I25A-QP and Gα_q_-I25A-RC mutants (Fig. 1, *C* and *D*). The effects of the I25A mutants on MAPK activity were further validated with the serum response element (SRE) luciferase reporter assay, which monitors both Rho and MAPK-dependent activity (Fig. 1*E*). As indicated, the CA Gα_q_-QL mutant robustly stimulated SRE-dependent luciferase activity. Introduction of the I25A mutation into Gα_q_-QL (Gα_q_-I25A-QL) strongly inhibited the ability to stimulate SRE luciferase activity, similar to the lack of signaling by the Gα_q_-QL-C9,10S palmitoylation-deficient mutant and the loss of signaling upon treatment of cells expressing Gα_q_-QL with YM. As expected, the CA Gα_q_-QP and Gα_q_-RC mutants also robustly stimulated SRE luciferase activity, while the Gα_q_-I25A-QP and Gα_q_-I25A-RC mutants completely failed to stimulate luciferase activity, similar to cells treated with YM (Fig. 1*E*). The decreased signaling by Gα_q_-I25A-QL in comparison to Gα_q_-QL is much more drastic in the SRE luciferase assay than the pERK immunoblot assay (Fig. 1, *C* and *D*). Although this is somewhat surprising since the SRE luciferase readout is downstream of the MAPK pathway, we note that the SRE luciferase assay provides a very high signal-to-noise readout for Gα_q_-QL signaling (>100-fold above basal vector alone or WT Gα_q_) and is consistently very sensitive to disruption of Gα_q_-QL signaling (*e.g.*, Gα_q_-I25A-QL only signals 5-6-fold above basal). Taken together, these results indicate that the introduction of the I25A mutation to disrupt binding to Gβγ inhibited oncogenic signaling of the CA Gα_q_ mutants through the YAP and MAPK pathways, and the Gα_q_-QP and Gα_q_-RC mutants are more sensitive to the I25A mutation than Gα_q_-QL.

### Differential association with Gβγ by CA Gα_q_ and Gα_q_-I25A mutants

**Figure 2:**
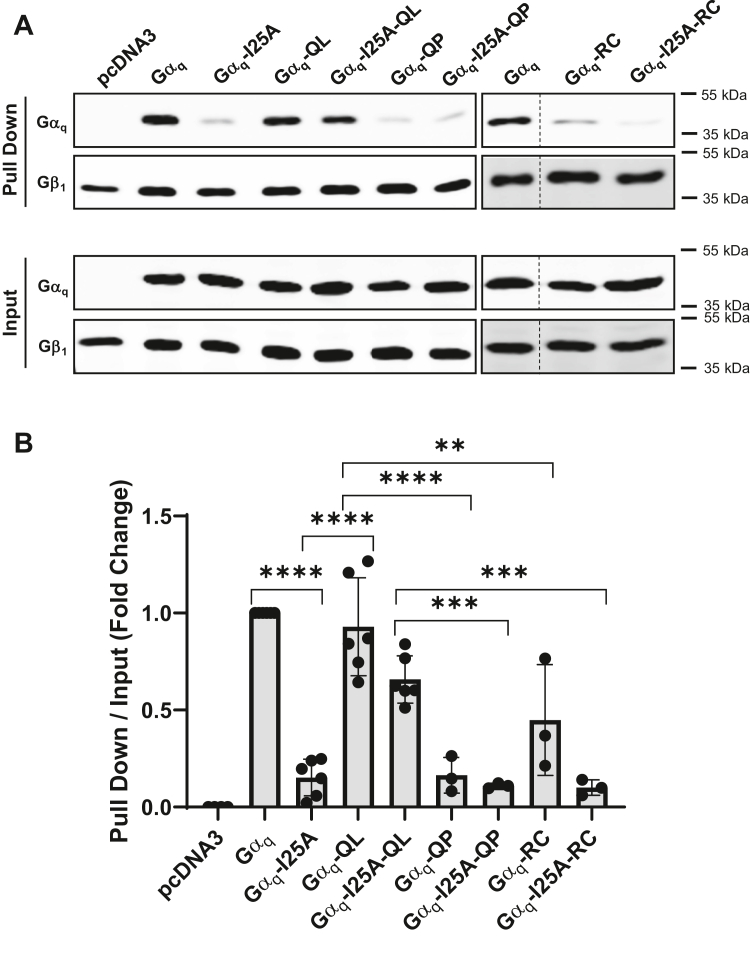
**Constitutively active Gα**_**q**_**and Gα**_**q**_**-I25A mutants have differential association with Gβγ.***A* and *B*, HEK 293 6x-His-β_1_γ_2_ stable cells were transfected with pcDNA3, WT Gα_q_, Gα_q_-I25A, Gα_q_-QL, Gα_q_-I25A-QL, Gα_q_-QP, Gα_q_-I25A-QP, Gα_q_-RC, or Gα_q_-I25A-RC. The cells were lysed, and an Ni-NTA pull-down assay was performed, as described under Experimental procedures. *A*, pull down and input lysates were immunoblotted using antibodies for the proteins indicated. *B*, the Gα_q_ pull-down signal intensities were quantified and normalized to the respective Gα_q_ input signal intensity. Results are shown as mean ± SD. Statistical significance is indicated (n = 6 for pcDNA3, WT Gα_q_, Gα_q_-I25A, Gα_q_-QL, Gα_q_-I25A-QL; n = 3 for Gα_q_-QP, Gα_q_-I25A-QP, Gα_q_-RC, or Gα_q_-I25A-RC, ∗∗*p* < 0.01; ∗∗∗*p* < 0.005; ∗∗∗∗*p* < 0.0001, two-way ANOVA, Šidák's multiple comparison’s test).

Previous studies in our lab have elucidated that CA Gα_q_-QL has an increased association with Gβγ compared to Gα_q_-RC (35). Furthermore, we have also shown that Gα_q_-I25A-QL maintains a substantial association with Gβγ compared to an almost complete loss of Gβγ association when the I25A mutation is introduced into WT Gα_q_ or Gα_q_-RC (35). It has also been previously established that Gα_q_-QP has reduced binding to effector proteins, such as p63RhoGEF, Trio, and GRK2 (40). Thus, we wanted to further characterize the interactions between Gβγ and CA Gα_q_-QL/P and Gα_q_-RC and the corresponding I25A mutants. To study this, we transiently transfected the WT or CA Gα_q_ and I25A mutants into HEK293 cells which stably expressed 6x-His-Gβ_1_y_2_ and pulled down Gβ_1_ using Ni-NTA beads. The relative association of Gα_q_ bound to Gβ_1_γ_2_ was revealed through immunoblotting (Fig. 2*A*) and was further quantified (Fig. 2*B*). As expected, WT Gα_q_ displayed a strong pull down with Gβ_1_γ_2_, but the introduction of the I25A mutation into WT Gα_q_ strongly decreased this interaction, indicating that the I25A mutation disrupts binding between Gα_q_ and Gβγ. Interestingly, Gα_q_-QL had a similar binding association to Gβ_1_γ_2_ with that of WT Gα_q_, and Gα_q_-QL bound significantly stronger to Gβ_1_γ_2_ than Gα_q_-QP and Gα_q_-RC (Fig. 2, *A* and *B*). Furthermore, Gα_q_-I25A-QL was more strongly bound to Gβ_1_γ_2_ than Gα_q_-I25A-QP and Gα_q_-I25A-RC (Fig. 2, *A* and *B*). We did not see a statistically significant difference in association with Gβγ between Gα_q_-QP and Gα_q_-RC and their corresponding I25A mutants. This is likely due to the low initial binding to Gβγ with both Gα_q_-QP and Gα_q_-RC. Although we previously demonstrated the surprising ability of Gα_q_-QL to interact strongly with Gβγ and the poor interaction of Gα_q_-RC with Gβγ, we now show a dramatically decreased association of Gα_q_-QP with Gβγ compared to Gα_q_-QL, even though both have a mutation of Q209. This differential binding to Gβγ between the CA Gα_q_ mutants likely contributes to the varying sensitivity in oncogenic signal disruption between the I25A mutants (Fig. 1).

### Expression of Gα_o_ to sequester endogenous Gβγ inhibits signaling by Gα_q_-QL/P and Gα_q_-RC

**Figure 3:**
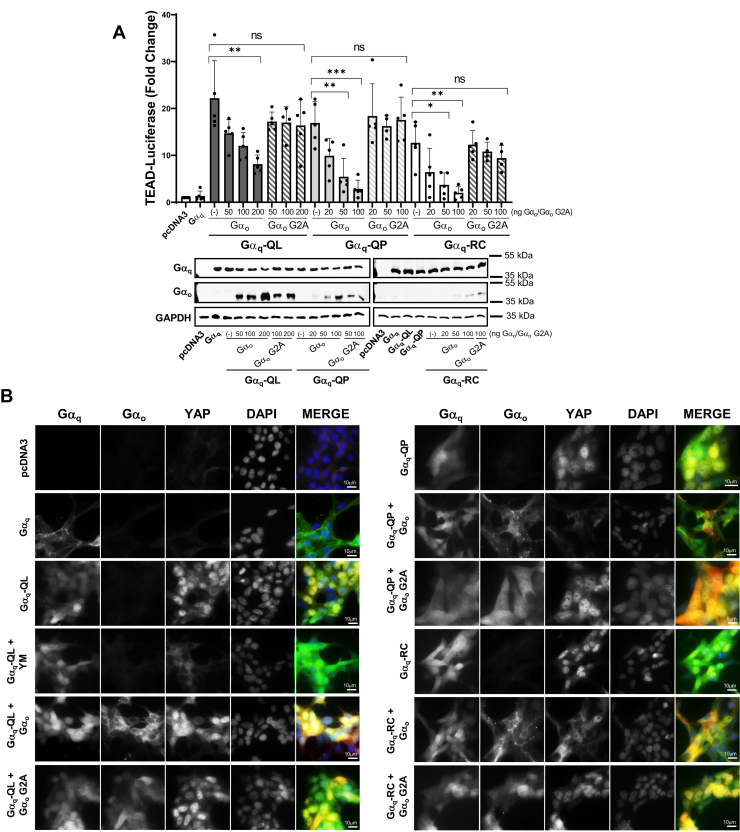
**Gα**_**o**_**expression to bind Gβγ inhibits YAP signaling from constitutively active Gα**_**q**_**mutants.***A*, HEK 293 Gα_q/11_ KO cells were transfected with pcDNA3, WT Gα_q_, Gα_q_-QL, Gα_q_-QP, or Gα_q_-RC, along with 8x-GTIIC Luciferase and Renilla control plasmids and along with varying amounts of Gα_o_ or Gα_o_-G2A expression plasmids. Cell lysates were prepared and luciferase assays were performed and quantified. Graph indicates fold change over pcDNA3. Results are shown as mean ± SD. Statistical significance is indicated (n = 4 or 5, ∗*p* < 0.05; ∗∗*p* < 0.01; ∗∗∗*p* < 0.005, two-way ANOVA, Šidák's multiple comparison’s test). Lysates were immunoblotted for Gα_q_, Gα_o_, and GAPDH protein levels. *B*, HEK 293 Gα_q/11_ KO cells were transfected with pcDNA3, WT YFP-Gα_q_, YFP-Gα_q_-QL, YFP-Gα_q_-QP, or YFP-Gα_q_-RC, along with 250 ng of Gα_o_ or Gα_o_ G2A. Cells indicated with Gα_q_-QL + YM were transfected with YFP-Gα_q_-QL and treated with 1 μM YM for 16 h. YFP-Gα_q_, Gα_o_, YAP, and nuclei (DAPI) stains were imaged *via* immunofluorescence microscopy (n = 3). YAP, Yes-associated protein.

Considering that inhibiting the association between the CA Gα_q_ mutants and Gβγ with the N-terminal I25A mutation significantly inhibited oncogenic signaling, we wanted to explore other methods of disrupting the interaction between Gβγ and CA Gα_q_. Overexpression of Gα_i_ family proteins has been previously used to sequester endogenous Gβγ in cells (41, 42, 43). Thus, we overexpressed Gα_o_, a Gα_i_ family member, with the CA Gα_q_-QL/QP and Gα_q_-RC mutants to bind and sequester endogenous Gβγ to ultimately decrease or prevent binding of endogenous Gβγ to the expressed Gα_q_. We also used a Gα_o_ G2A mutant as a negative control. The G2A mutation prevents both myristoylation and palmitoylation; this renders Gα_o_ cytoplasmic and poorly able to bind Gβγ due to its inability to localize to cellular membranes (44). YAP activity was monitored using the TEAD luciferase reporter assay with cotransfection of the CA Gα_q_ mutants and increasing amounts of the Gα_o_ expression plasmids (Fig. 3*A*). Interestingly, cotransfection with 200 ng of the Gα_o_ DNA was required to decrease Gα_q_-QL stimulation of TEAD luciferase activity by 63%. In comparison, cotransfection with 100 ng of Gα_o_ plasmid DNA achieved 83% and 84% reduction in signaling by Gα_q_-QP and Gα_q_-RC, respectively. Transfection with the control Gα_o_ G2A constructs did not reduce the luciferase reporter activity in response to the CA Gα_q_ mutants (Fig. 3*A*). The ability of the expression of Gα_o_ to reduce CA Gα_q_-stimulated YAP activity was also monitored through nuclear localization of YAP by immunofluorescence microscopy. Gα_o_ localized strongly at cellular membranes, while the Gα_o_ G2A mutant was primarily cytoplasmic, indicating the expected localization of the proteins. Strong nuclear YAP was detected with the CA Gα_q_-QL, Gα_q_-QP, and Gα_q_-RC mutants, indicating stimulation of the YAP pathway (Fig. 3*B*). As expected, expression of Gα_o_ G2A with the CA Gα_q_ mutants did not disrupt nuclear localization of YAP. Overexpression of Gα_o_ inhibited YAP nuclear localization stimulated by Gα_q_-QP and Gα_q_-RC (Fig. 3*B*). Conversely, cotransfection of Gα_o_ with Gα_q_-QL failed to prevent strong YAP nuclear localization (Fig. 3*B*), further suggesting that the Gα_q_-QP and Gα_q_-RC mutants are more sensitive to Gβγ binding disruption than Gα_q_-QL.

**Figure 4:**
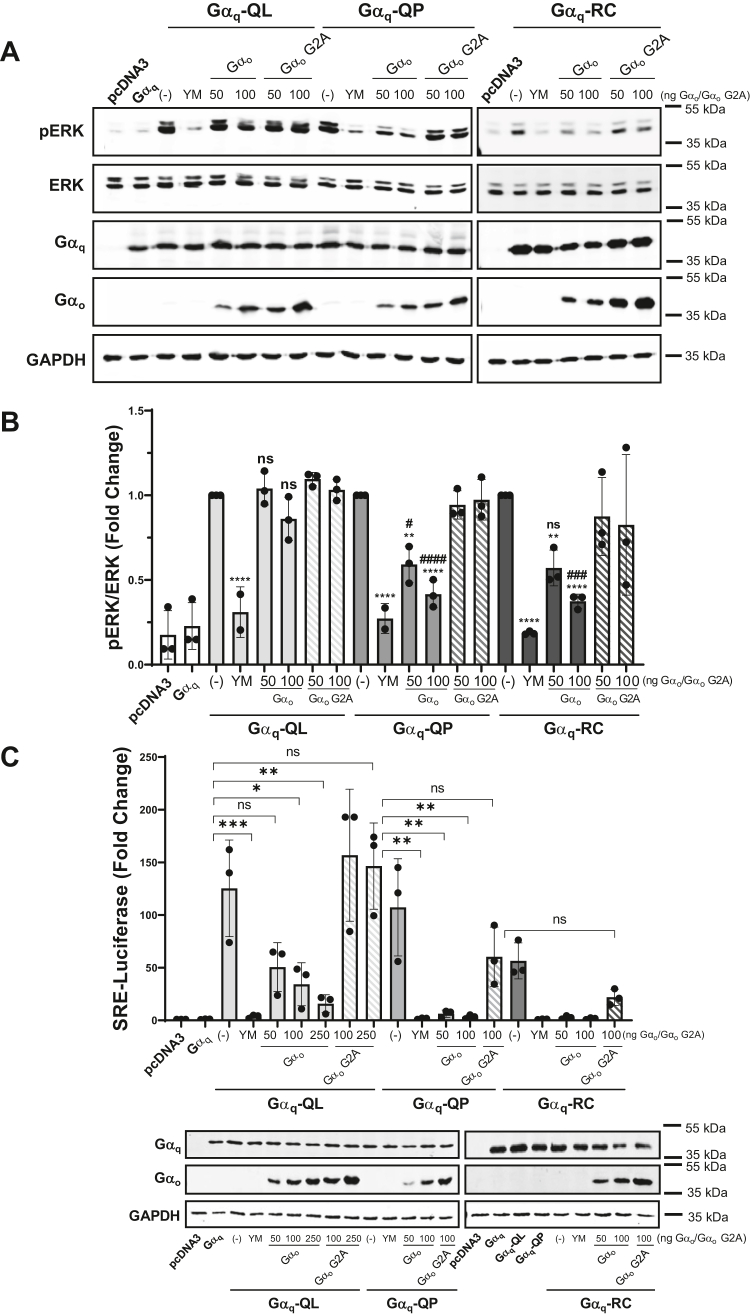
**Constitutively active Gα**_**q**_**-dependent MAPK signaling is inhibited by overexpression of Gα**_**o**_**.***A* and *B*, HEK 293 Gα_q/11_ KO cells were transfected with pcDNA3, WT Gα_q_, Gα_q_-QL, Gα_q_-QP, or Gα_q_-RC, along with 50 ng or 100 ng of Gα_o_ or Gα_o_-G2A. YM indicates expression of Gα_q_-QL, Gα_q_-QP, or Gα_q_-RC with treatment with 1 μM YM for 16 h. *A*, cell lysates were prepared and immunoblotted for pERK, ERK, Gα_q_, Gα_o_, and GAPDH. *B*, pERK/ERK signal intensities were quantified. Graph indicates pERK/ERK signal intensities normalized to Gα_q_-QL, Gα_q_-QP, or Gα_q_-RC without Gα_o_ expression. Results are shown as mean ± SD. Statistical significance is indicated. (∗ indicates significance between Gα_q_-QL, Gα_q_-QP, or Gα_q_-RC and treatment with YM or Gα_o_; # indicates significance between treatment with Gα_o_ and corresponding concentration of Gα_o_ G2A) (n = 3; n = 2 for Gα_q_-QL and Gα_q_-QP with YM treatment, ∗*p* < 0.05; ∗∗*p* < 0.01; ∗∗∗*p* < 0.005; ∗∗∗∗*p* < 0.0001, two-way ANOVA, Šidák's multiple comparison’s test). *C*, HEK 293 Gα_q/11_ KO cells were transfected with pcDNA3, WT Gα_q_, Gα_q_-QL, Gα_q_-QP, or Gα_q_-RC, along with varying concentrations of Gα_o_ or Gα_o_ G2A. YM indicates expression of Gα_q_-QL, Gα_q_-QP, or Gα_q_-RC with 1 μM treatment of YM for 16 h. Cell lysates were prepared and luciferase assays were performed and quantified. Results are shown as mean ± SD. Graph indicates fold change over pcDNA3. Statistical significance is indicated (n = 3; ∗*p* < 0.05; ∗∗*p* < 0.01; ∗∗∗*p* < 0.005, two-way ANOVA, Šidák's multiple comparison’s test). Lysates were immunoblotted for Gα_q_, Gα_o_, and GAPDH protein levels. MAPK, mitogen-activated protein kinase; pERK, phospho-ERK.

To monitor the effects of Gα_o_ expression and subsequent Gβγ sequestration on CA Gα_q_-promoted MAPK activity, we immunoblotted for pERK levels (Fig. 4, *A* and *B*). The expression of the CA Gα_q_ mutants resulted in strong stimulation of pERK. Expression of Gα_o_ resulted in no significant decrease in pERK activity for Gα_q_-QL. However, cotransfection of Gα_q_-QP or Gα_q_-RC with 50 ng and 100 ng of Gα_o_ plasmid resulted in a strong decrease in pERK activity (Fig. 4, *A* and *B*). There was no significant change in pERK levels with the Gα_o_ G2A control (Fig. 4, *A* and *B*). These results were further validated using the SRE luciferase reporter assay (Fig. 4*C*). Expression of Gα_q_-QL, Gα_q_-QP, and Gα_q_-RC robustly activated SRE-dependent luciferase activity, while treatment with YM abolished luciferase activity. Consistent with results from the other signaling assays (Figs. 3 and 4, *A* and *B*), cotransfection with 50 ng Gα_o_ plasmid was sufficient to completely abolish Gα_q_-QP– and Gα_q_-RC–stimulated SRE luciferase activity, while up to 250 ng of Gα_o_ plasmid was required for near complete inhibition of Gα_q_-QL–stimulated SRE luciferase activity (Fig. 4*C*). These findings indicate that the expression of Gα_o_ to sequester endogenous Gβγ inhibits the signaling activity of Gα_q_-QL/P and Gα_q_-RC. The results also further validate that the Gα_q_-QL CA mutant is less sensitive to signaling disruption than the Gα_q_-QP and Gα_q_-RC mutants.

### Gα_o_ expression inhibits the binding of Gβγ to CA Gα_q_ mutants

**Figure 5:**
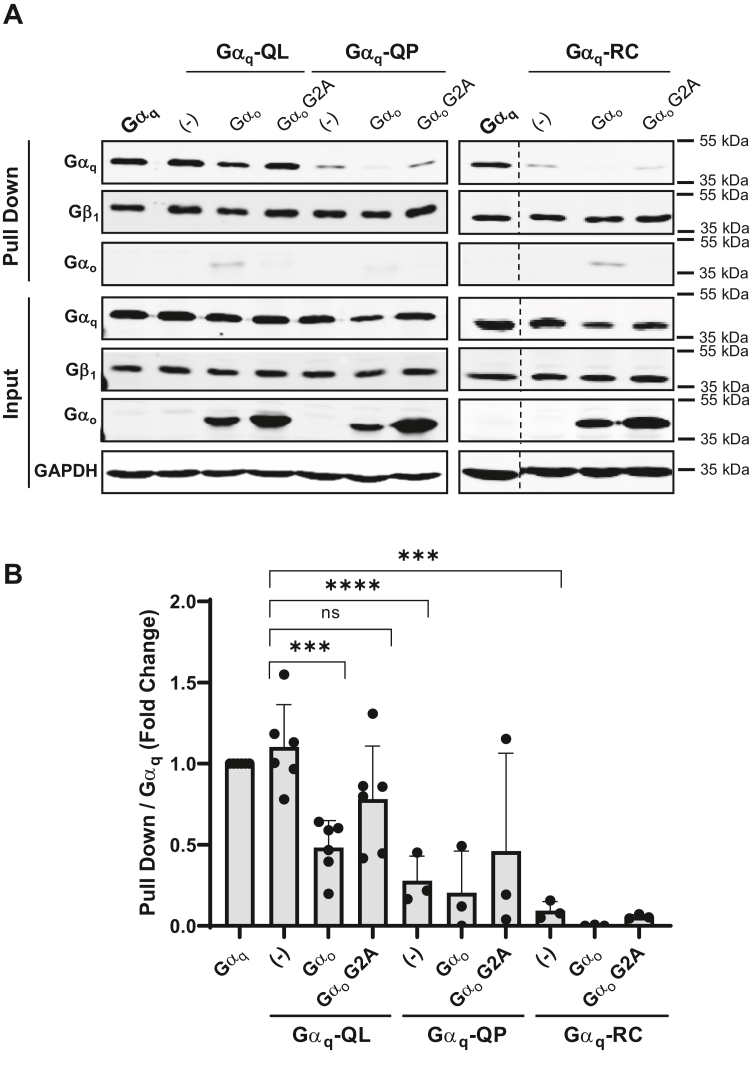
**Gα**_**o**_**expression disrupts Gβγ binding to constitutively active Gα**_**q**_**mutants.***A* and *B*, HEK 293 6x-His-β_1_γ_2_ stable cells were transfected with WT Gα_q_, Gα_q_-QL, Gα_q_-QP, or Gα_q_-RC, along with 300 ng of Gα_o_ or Gα_o_ G2A. The cells were lysed, and an Ni-NTA pull-down assay was performed. *A*, pull down and input lysates were immunoblotted using antibodies for the proteins indicated. *B*, the Gα_q_ pull down signal intensities were quantified and normalized to WT Gα_q_ pull-down signal intensity. Results are shown as mean ± SD. Statistical significance is indicated (n = 3; n = 6 for WT Gα_q_, Gα_q_-QL, and Gα_q_-QL plus Gα_o_ or Gα_o_ G2A, ∗∗∗*p* < 0.005; ∗∗∗∗*p* < 0.0001, two-way ANOVA, Šidák's multiple comparison’s test).

Next, we wanted to confirm that the expression of Gα_o_ could sequester Gβγ and inhibit the binding of Gβγ to Gα_q_-QL/P and Gα_q_-RC. To examine this, we coexpressed Gα_o_ or Gα_o_ G2A with the CA Gα_q_ mutants in 6x-His-Gβ_1_y_2_ HEK 293 stable cells and pulled down Gβ_1_γ_2_ with Ni-NTA beads. Immunoblots were used to detect Gα_q_ and Gα_o_ bound to Gβ_1_ (Fig. 5, *A* and *B*). As indicated in Figure 2, Gα_q_-QL had a stronger binding association with Gβ_1_ than Gα_q_-QP and Gα_q_-RC. Gα_o_ was bound to Gβ_1_ in the pull-down extract, but Gα_o_ G2A had minimal to no interaction with Gβ_1_ as expected. The expression of Gα_o_ resulted in significantly decreased binding of Gα_q_-QL to Gβ_1_ (Fig. 5, *A* and *B*). Expression of Gα_o_ consistently resulted in a noticeable decrease in the pull down of Gα_q_-QP and Gα_q_-RC with Gβγ, but this did not reach statistical significance due to the already low level of Gα_q_-QP and Gα_q_-RC association with Gβγ in the absence of Gα_o_ expression. These findings confirm that overexpression of Gα_o_ sequesters Gβγ and prevents binding of Gα_q_-QL/P and Gα_q_-RC to Gβγ.

### Depletion of Gβ_1/2_ inhibits oncogenic signaling by Gα_q_-Q209P in UM cell lines

**Figure 6:**
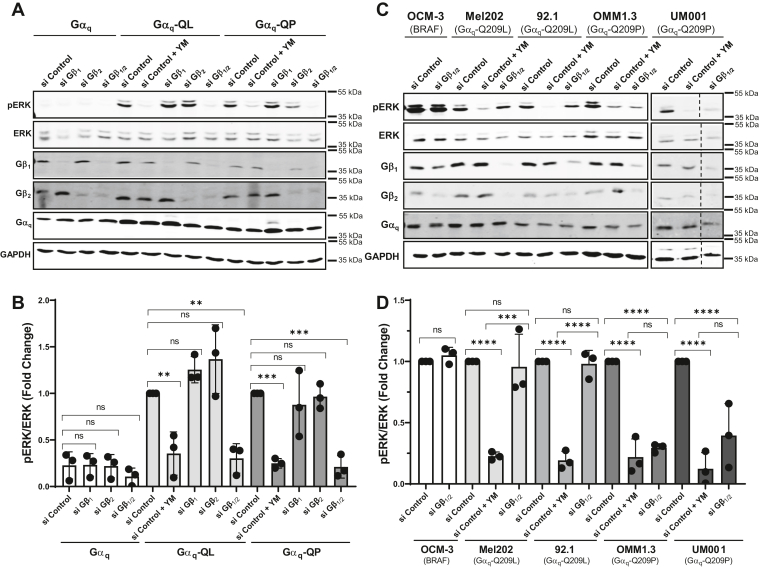
**Knockdown of Gβ**_**1/2**_**inhibits oncogenic MAPK signaling in uveal melanoma cells with Gα**_**q**_**-Q209P mutation.***A* and *B*, HEK 293 Gα_q/11_ KO cells were transfected with control, Gβ_1_, Gβ_2_, or Gβ_1/2_ siRNA. After 24 h, cells were transfected with WT Gα_q_, Gα_q_-QL, or Gα_q_-QP. YM indicates treatment with 1 μM YM for 16 h. *A*, cell lysates were prepared and immunoblotted for pERK, ERK, Gβ_1_, Gβ_2_, Gα_q_, and GAPDH. *B*, pERK/ERK signal intensities were quantified. Results are shown as mean ± SD. Graph indicates pERK/ERK signal intensities normalized to Gα_q_-QL or Gα_q_-QP with control siRNA. Statistical significance is indicated (n = 3; ∗∗*p* < 0.01; ∗∗∗*p* < 0.005; two-way ANOVA, Tukey's multiple comparison’s test). *C* and *D*, OCM-3, Mel202, 92.1, OMM1.3, or UM001 cells were transfected with control or Gβ_1/2_ siRNA. YM indicates treatment with 1 μM YM for 16 h. *C*, cell lysates were prepared and immunoblotted for pERK, ERK, Gβ_1_, Gβ_2_, Gα_q_, and GAPDH. *D*, pERK/ERK signal intensities were quantified. Results are shown as mean ± SD. Graph indicates pERK/ERK signal intensities normalized to each cell line with control siRNA. Statistical significance is indicated (n = 3, ∗∗∗*p* < 0.005; ∗∗∗∗*p* < 0.0001, two-way ANOVA, Tukey's multiple comparison’s test). MAPK, mitogen-activated protein kinase; pERK, phospho-ERK.

Our studies demonstrate that disrupting the interaction between Gβγ and CA Gα_q_ through the N-terminal I25A mutation or expression of Gα_o_ is sufficient to inhibit overactive Gα_q_ signaling in HEK 293 Gα_q/11_ KO cells. We next wanted to determine if inhibiting Gβγ binding to CA Gα_q_ would be sufficient to disrupt oncogenic signaling in UM cells containing the Gα_q_-QL or Gα_q_-QP mutants. To study this, we depleted Gβ_1_ and Gβ_2_, two predominant Gβ subunits, through siRNA transfections. Sufficient knockdown of Gβ_1_ and Gβ_2_ was first validated in HEK 293 Gα_q/11_ KO cells (Fig. 6, *A* and *B*). siRNA molecules specific for Gβ_1_, Gβ_2_, and both Gβ_1_ and Gβ_2_ were transfected into the HEK 293 Gα_q/11_ KO cells, and WT Gα_q_, Gα_q_-QL, or Gα_q_-QP constructs were transfected after 24 h. pERK levels were detected and quantified through immunoblotting (Fig. 6, *A* and *B*). The results indicate that siRNA depletion of Gβ_1/2_ significantly disrupted pERK activity driven by both Gα_q_-QL and Gα_q_-QP (Fig. 6, *A* and *B*). Gβ_1_ or Gβ_2_ siRNA knockdown alone was insufficient to significantly inhibit pERK activation (Fig. 6, *A* and *B*). Considering the effectiveness of Gβ_1/2_ depletion on pERK activity in the HEK 293 Gα_q/11_ KO cells, we decided to knock down Gβ_1/2_ in four UM cell lines: 1) Mel202 (Gα_q_-Q209L mutation), 2) 92.1 (Gα_q_-Q209L mutation), 3) OMM1.3 (Gα_q_-Q209P mutation), and 4) UM001 (Gα_q_-Q209P mutation). We also used the OCM-3 cell line as a control. The oncogenic activity of OCM-3 cells is driven by mutant BRAF (V600E) as opposed to mutant Gα_q/11_; thus, we would expect Gβ_1/2_ depletion to have minimal effect on pERK activity (45). All cell lines were treated with control or Gβ_1/2_ siRNA for 96 h. Mel202, 92.1, OMM1.3, and UM001 cells were also treated with YM as a control. pERK levels were immunoblotted (Fig. 6*C*) and quantified (Fig. 6*D*). Interestingly, the OMM1.3 and UM001 cell lines which contain the Gα_q_-Q209P mutation had significantly decreased pERK levels with Gβ_1/2_ knockdown, similar to that of YM treatment (Fig. 6, *C* and *D*). There was no significant difference in pERK levels with Gβ_1/2_ depletion in the Mel202 and 92.1 cells which contain the Gα_q_-Q209L mutation (Fig. 6, *C* and *D*). This data further suggests that there is differential sensitivity to the inhibition of oncogenic signaling between cell lines with the Gα_q_-Q209L and Gα_q_-Q209P mutations.

## Discussion

The results presented in this study demonstrate the importance of Gβγ for signaling by CA mutants of Gα_q_ and show a surprising differential sensitivity of CA Gα_q_ mutants to the inhibition of signaling upon disruption of their interaction with Gβγ. We used two methods to disrupt Gβγ interaction with CA Gα_q_—introduction of a Gβγ-binding disrupting mutation, I25A, into CA Gα_q_ and sequestration of endogenous Gβγ by expression of Gα_o_—to show that inhibiting interaction with Gβγ can drastically reduce CA Gα_q_-stimulated signaling to both the MAPK and YAP/TAZ pathways. Moreover, signaling by CA Gα_q_-Q209P, as well as CA Gα_q_-R183C, was much more effectively prevented by Gβγ binding disruption than signaling by CA Gα_q_-Q209L. Lastly, we extended the analysis to UM cell lines containing either the oncogenic driver mutant Gα_q_-Q209L or Gα_q_-Q209P and showed that depletion of Gβ_1/2_ strongly inhibited activation of the MAPK pathway in cells with the Gα_q_-Q209P mutant but not in cells with the Gα_q_-Q209L. Our results suggest that disrupting the interaction between Gβγ and CA Gα_q_ may be a novel approach for inhibiting aberrant Gα_q/11_ signaling.

Although the importance of Gβγ for localization and GPCR-dependent activation of WT, GDP-bound Gα subunits is clearly established, the idea that activated, GTP-bound Gα subunits also require Gβγ to carry out their cellular functions remains poorly defined. Classically, it is generally considered that upon GPCR stimulation, GTP is exchanged for GDP on the Gα subunit, and Gβγ dissociates from Gα. Therefore, if the Gα subunit is “locked” in a GTP-bound state, as would be the case for the GTPase-deficient CA Gα_q_ mutants studied herein, it would be expected that CA Gα mutants would not retain binding to Gβγ. However, several studies have challenged the classical heterotrimer dissociation model, indicating that at least some Gα subunits maintain association with Gβγ when they are in the active, GTP-bound state due to the presence of a GTPase-inhibiting mutation or in response to GPCR activation (46, 47). The studies presented here using Gβγ pull-down assays (Figs. 2 and 4), along with previous work in our lab and others (35, 36), support the idea that the CA mutant Gα_q_-QL retains binding to Gβγ, similar to that of WT Gα_q_. Our data also indicate that CA Gα_q_-QP and Gα_q_-RC can bind Gβγ, although to a much lesser extent than Gα_q_-QL. Gβγ pull-down assays show a low level of Gα_q_-QP and Gα_q_-RC associated with Gβγ, but this pull-down of Gα_q_-QP and Gα_q_-RC is decreased by the introduction of the I25A mutation or expression of Gβγ-sequestering Gα_o_ (Figs. 2 and 4). Support for the binding of Gβγ to Gα_q_-QP and Gα_q_-RC is provided further by our data showing an almost complete loss of signaling by Gα_q_-QP and Gα_q_-RC when they contain the I25A mutation or when Gα_o_ is expressed (Figs. 1, 3 and 4). Importantly, the interaction of CA Gα with Gβγ is not unique to Gα_q_. Our previous work showed that CA Gα_s_ mutants, such as Gα_s_-QL and Gα_s_-RC, also retain binding to Gβγ (35). More recent work in the field indicates that, along with Gα_q_-QL, Gα_13_-QL also maintains a robust binding association with Gβγ; however, Gα_i_-QL does not associate with Gβγ, highlighting an intriguing specificity (36).

A structural view of how CA Gα_q_ mutants interact with Gβγ and a clear understanding of how Gβγ regulates constitutive signaling by CA Gα_q_ remain elusive. Crystal structures indicate that the inactive, GDP-bound Gα subunit has two main points of contact with Gβγ: 1) a central switch region, which undergoes a conformational change upon GTP binding and 2) an N-terminal α helical domain (32, 33, 34). GTP binding–dependent conformational changes in the Gα switch regions result in a decreased affinity for Gβγ; however, this appears not to be sufficient for complete disruption of Gα and Gβγ association in some cases. Thus, the Gα N-terminus may play an essential role in maintaining association with Gβγ when Gα is placed in an active conformation by GPCR activation or through the presence of a GTPase-inhibiting mutation. Previous work showing that the N-terminus is essential for Gα interaction with Gβγ and that discrete mutations in the N-termini of several Gα, including Gα_q_ and Gα_s_, are sufficient to disrupt interaction with Gβγ support this model (27, 48, 49, 50, 51, 52). Furthermore, a recent study comparing the ability of Gα_q_-QL and Gα_13_-QL to associate with Gβγ with the inability of Gα_i_-QL to associate with Gβγ showed that a Gα_i/13_-QL chimera, in which the N-terminal helix of Gα_13_-QL is replaced with the N-terminal helix of Gα_i_-QL, failed to interact with Gβγ (36). Thus, a clamshell model has been put forth in which GTP-bound Gα and Gβγ “open up” but remain associated through the N-terminus of Gα (46, 47). Nonetheless, this model does not fully explain why Gα_q_-QP and Gα_q_-RC weakly associate with Gβγ in pull-down assays since Gα_q_-QL, Gα_q_-QP, and Gα_q_-RC have identical N-termini. Intriguingly, in G protein heterotrimer crystal structures (32, 33), Q209 in Gα_q_ and the corresponding glutamine in Gα_t_ make contact with Gβ, raising the possibility that the leucine at this position in Q209L promotes interaction with Gβ, either directly or indirectly by effecting a conformational change in Gβ-interacting switch regions. Clearly, future work is needed to understand how activated Gα, such as Gα_q_-QL, retain association with Gβγ. The second question of why certain activated Gα need to interact with Gβγ is likewise not well-understood. Association with Gβγ is critical for palmitoylation and membrane localization of Gα_q_, and it is known that Gα_q_ cannot signal when it is not palmitoylated and not membrane-localized (27). A complete lack of signaling by Gα_q_-QL-C9,10S, in which the two sites of palmitoylation are mutated, reinforces the requirement for palmitoylation for Gα_q_ signaling (Fig. 1, *A* and *E*). Thus, one reason why CA Gα_q_ mutants in this study showed decreased or loss of signaling to the MAPK and YAP pathways when interaction with Gβγ is disrupted is a failure to be efficiently palmitoylated and membrane-bound. Indeed, Gα_q_-I25A-QL retains stronger plasma membrane localization than Gα_q_-I25A-QP and Gα_q_-I25A-RC (Fig. S1). Additional roles likely exist for the continued association of CA Gα_q_ with Gβγ. Notably, our previous work showed that Gα_q_-I25A-RC failed to stimulate IP production, consistent with the lack of MAPK and YAP pathway activation observed here, and the introduction of a site for N-terminal myristoylation into Gα_q_-I25A-RC to recover palmitoylation and membrane binding failed to recover IP signaling, demonstrating that enhanced membrane localization is not sufficient to overcome the disruption of association with Gβγ (35). Continued association of CA Gα_q_ with Gβγ may allow both components to simultaneously activate an effector, such as PLC-β; such dual activation of a single effector may be required for efficient signaling in specific contexts. In addition, a recent study showing a strong association of Gα_q_-QL and Gα_13_-QL with Gβγ provided evidence for a model in which retained association with Gβγ prevents Gβγ-mediated P-REX1-Rac1 signaling while still allowing Gα_q_-QL and Gα_13_-QL to interact with their effectors (36). Moreover, we cannot rule out a role for GPCR stimulation in signaling by the Gα_q_ mutants and thus a potential role for Gβγ in facilitating GPCR coupling. There are likely to be multiple functional consequences of maintained association of CA Gα_q_ with Gβγ, and our work here provides a strong demonstration of the importance of retaining interaction with Gβγ for the activation of oncogenic signaling pathways by the Gα_q_-QL, Gα_q_-QP, and Gα_q_-RC mutants that are drivers of UM.

The most novel and striking finding in our studies was that the CA Gα_q_-QP and Gα_q_-RC mutants are dramatically more sensitive to Gβγ binding disruption than Gα_q_-QL. This difference was demonstrated with the introduction of the I25A mutation, expression of Gα_o,_ and siRNA depletion of Gβ_1/2_. In all signaling assays tested, the introduction of I25A into Gα_q_-QL to generate Gα_q_-I25A-QL only partially reduced signaling compared to Gα_q_-QL (Fig. 1). On the other hand, Gα_q_-I25A-QP and Gα_q_-I25A-RC were completely deficient in signaling as measured by TEAD- and SRE-luciferase reporter assays and as detected by YAP localization (Fig. 1, *A*, *B* and *E*), although only partial loss of signaling was observed in when measuring pERK (Fig. 1, *C* and *D*). Expression of the increasing amounts of Gα_o_ to sequester Gβγ provided a powerful tool to demonstrate titratable differences in sensitivity to disruption of Gβγ binding to the CA Gα_q_ mutants (Figs. 3 and 4). In the TEAD-luciferase assay, transfection with 200 ng of Gα_o_ expression plasmid resulted in 63% inhibition of Gα_q_-QL, but transfection with 100 ng of Gα_o_ plasmid reduced the signaling of Gα_q_-QP and Gα_q_-RC by 83% and 84%, respectively (Fig. 3*A*). Using the SRE-luciferase assay, transfection with 50 ng of Gα_o_ expression plasmid was sufficient to completely abolish signaling of Gα_q_-QP and Gα_q_-RC, while transfection with 250 ng of Gα_o_ plasmid was required to observe a similar almost-complete inhibition of Gα_q_-QL signaling (Fig. 4*C*). Likewise, transfection with 50 or 100 ng of Gα_o_ plasmid failed to inhibit Gα_q_-QL–stimulated pERK levels, but transfection with 50 or 100 ng of Gα_o_ plasmid provided significant inhibition of Gα_q_-QP– and Gα_q_-RC–stimulated pERK (Fig. 4, *A* and *B*). Gβγ pull-down experiments showed the interaction of Gα_q_-QL with Gβγ, similar to the levels of WT Gα_q_ interaction with Gβγ, and, importantly, the I25A mutation and expression of Gα_o_ only partially disrupted the interaction (Figs. 2 and 5); these results thus provided a mechanistic interpretation for the inability to fully inhibit Gα_q_-QL signaling when Gβγ binding is disrupted. The much lower levels of association with Gβγ of Gα_q_-QP and Gα_q_-RC, compared to Gα_q_-QL, can explain the clear differences in sensitivity to the inhibition of signaling upon disruption of Gβγ binding. Gα_q_-QL has a stronger association with Gβγ, which ultimately promotes resistance to the disruption of aberrant cell signaling.

The results presented here are the first to show that signaling by Gα_q_-QL *versus* Gα_q_-QP can be differentially disrupted. They both contain a single amino acid substitution at Q209, yet exhibit dramatic differences in the ability of their signaling to be inhibited by disruption of the interaction with Gβγ (Figs. 1, 3 and 4). Interestingly, a recent report compared Gα_q_-QL and Gα_q_-QP and found no difference in their signaling ability (40). We confirmed here using multiple signaling assays that Gα_q_-QL and Gα_q_-QP constitutively activated signaling pathways to a similar degree upon similar protein expression levels (Figs. 1, 3 and 4). However, Maziarz, *et al.* (40) showed that Gα_q_-QP compared to Gα_q_-QL displayed much lower levels of association with effectors and RGS proteins. It was also observed that whereas Gα_q_-QL showed the expected protein fragment in a trypsin protection assay, Gα_q_-QP did not, leading to the conclusion that Gα_q_-QP exists in a different active conformation compared to Gα_q_-QL (40). It seems likely that substitution of Q209 with proline changes the conformational dynamics compared to Gα_q_-QL, consistent with differing levels of binding to Gβγ (Figs. 2 and 5) and the consequent differences in the inhibition of signaling that we show here (Figs. 1, 3 and 4).

To further validate the differences in inhibition of Gα_q_-QL *versus* Gα_q_-QP, we used UM cells containing a Q209L or Q209P mutation in endogenous Gα_q_. We used siRNA to deplete endogenous Gβ_1_ and Gβ_2_ and analyzed constitutively activated levels of pERK (Fig. 6). Depletion of Gβ_1/2_ profoundly inhibited pERK activation in two cell lines, OMM1.3 and UM001, harboring Gα_q_-QP, equivalent to inhibition by treatment with the Gα_q_ inhibitor YM-254890. In contrast, no inhibition of pERK was seen upon Gβ_1/2_ depletion in two UM cell lines, Mel202 and 92.1, containing Gα_q_-QL, even though YM-254890 efficiently abolished pERK signaling in those cells. These results provide strong support for the greater sensitivity of Gα_q_-QP than Gα_q_-QL in terms of inhibition of oncogenic signaling by inhibiting interaction with Gβγ. Interestingly, when Gβ_1/2_ was depleted in HEK 293 Gα_q/11_ KO cells, signaling to pERK was prevented for both overexpressed Gα_q_-QL and Gα_q_-QP. We suspect that the difference in Gα_q_-QL sensitivity in HEK 293 Gα_q/11_ KO cells *versus* UM cells results from the differences in methods when examining the effect of Gβ_1/2_ knockdown on endogenous Gα_q_-QL compared to transfected Gα_q_-QL. Gβ_1/2_ depletion in HEK 293 Gα_q/11_ KO cells may prevent the subsequently transfected and thus newly synthesized Gα_q_-QL from ever localizing to membranes. In contrast, Gβ_1/2_ depletion in UM cells would have to disrupt endogenous Gα_q_-QL that is already at membranes and bound to Gβγ. Regardless, our results suggest that disrupting the interaction between Gβγ and CA Gα_q_ could be a novel therapeutic target in patients with UM, particularly those with the Q209P mutation. Approximately, 90% of patients with UM have mutations at Q209 and 5% have mutations at R183 in Gα_q/11_, causing constitutive activation of downstream signaling pathways. Mutations in Gα_q_ and Gα_11_ are mutually exclusive in UM, with Q209 mutations occurring with similar frequency in Gα_q_ and Gα_11_. Interestingly, in a study of 80 patient samples, 34% contained the Gα_q_-Q209P mutant, 13% contained the Gα_q_-Q209L mutant, and 43% harbored the Gα_11_-Q209L mutant; no samples with Gα_11_-Q209P were identified (8). Here, we show that disrupting the association between Gβγ and CA Gα_q_ disrupts oncogenic signaling involved in UM. Importantly, we suggest that the Gα_q_-QP and Gα_q_-RC are more sensitive to proliferative cell disruption. The importance of this novel differential sensitivity between the Gα_q_-QL and Gα_q_-QP mutations is highlighted by a recent retrospective analysis of 87 metastatic UM patients (53). The authors found no significant change in time from primary tumor diagnosis to liver metastasis between patients with the Gα_q/11_-QL or Gα_q/11_-QP mutations; however, patients with the Q209P mutation had significantly higher survival after metastasis than patients with the Q209L mutation. In particular, the median survival rate of patients with the Gα_q_-QL mutation was 21.5 months, while the survival rate after metastasis for patients with the Gα_q_-QP mutation was 35 months (53). We speculate that the differential binding between the Gα_q_-QL and Gα_q_-QP mutants with Gβγ and other effectors could potentially have an impact on oncogenicity and survival after metastasis.

## Experimental procedures

### Plasmids, siRNA, and reagents

HA-tagged WT Gα_q_, Gα_q_-QL, and Gα_q_-RC in pcDNA3 were described previously (35). The Q209P mutation for the Gα_q_-QP construct was generated from WT Gα_q_ *via* site-directed mutagenesis using the primers (Forward: 5′ – CGATGTAGGGGGCCCAAGGTCAGAGAGAAG – 3′, Reverse: 5′ – CTTCTCTCTGACCTTGGGCCCCCTACATCG – 3′). The Gα_q_-I25A, Gα_q_-I25A-QL, and Gα_q_-I25A-RC constructs were previously described (35). The I25A mutation was generated for Gα_q_-I25A-QP from Gα_q_-QP (Forward: 5′ – GCAGCTGCCGCTCGGCCTCGTCGTTGATCC – 3′, Reverse: 5′ – GGATCAACGACGAGGCCGAGCGGCAGCTGC – 3′). YFP-tagged Gα_q_ was provided by Catherine Berlot. Gα_o_ and Gα_o_-G2A plasmids were described previously (44). Lipofectamine 2000 used for Gα_q_ and Gα_o_ transient transfections was obtained from Invitrogen (Cat # 11668-019). siRNA targeting Gβ_1_ (GGAUAACAUUUGCUCCAUU), Gβ_2_ (ACUGGGUACCUGUCGUGUU), and both Gβ_1_ and Gβ_2_ (Gβ_1/2_) (ACGACGACUUCAACUGCAA) were previously described (54, 55). The ON-TARGET plus nontargeting siRNA (Horizon Discovery, Cat # D-001810-10-20) was used for control siRNA transfections. Lipofectamine RNAiMAX was used for siRNA transfections (Invitrogen, Cat # 13778-150). YM-254890 (YM) was obtained from Wako Chemicals (Cat # 257-00631).

### Antibodies

The antibodies for Gα_q_ (Cat #13927-1-AP) (for immunoblots with HEK 293 Gα_q/11_ KO lysates), GAPDH (Cat # 60004-1-Ig), and Gα_o_ (Cat # 12635-1-AP) were from Proteintech. The Gα_q_ (Cat # ab199533) (for immunofluorescence experiments), Gβ_1_ (Cat # ab137635), and Gβ_2_ (Cat # ab108504) antibodies were obtained from Abcam. The YAP antibody (Cat # sc-101199) was purchased from Santa Cruz. The Gα_q_ (Cat # 14373S) (for immunoblots with OCM-3, Mel202, 92.1, OMM1.3, and UM001 lysates), ERK (Cat # 4696S), pERK (Cat # 9101S), and Myc-tag (Cat # 2272S) antibodies were obtained from Cell Signaling Technologies. The HA-tag antibody 12CA5 was from Covance. The GRK4-6 antibody (Cat # 05-466) was obtained from Sigma-Aldrich. For immunofluorescence microscopy, the secondary antibodies Alexa Fluor 488 (goat anti-rabbit) (Cat # A-11034), Alexa Fluor 594 (goat anti-mouse) (Cat # A-11032), Alexa Fluor 594 (goat anti-rabbit) (Cat # A-11037), and Alexa Fluor 647 (goat anti-mouse) (Cat # A-32728) were purchased from Invitrogen. The secondary antibodies IRDye 680RD goat anti-rabbit IgG (H + L) (Cat # 92568071) and IRDye 800CW donkey anti-mouse IgG (H + L) (Cat # 92532212) were obtained from LI-COR and were used to visualize protein from all of the immunoblots.

### Cell culture and reagents

HEK 293 Gα_q/11_ KO cells were generously provided by Dr Asuka Inoue and were described previously (56). The HEK 293 Gα_q/11_ KO cells were cultured in Dulbecco’s modified Eagle’s medium (Corning, Cat # 10-017-CV) with 10% fetal bovine serum (FBS) (Gemini, Cat # 900-108) and 1% penicillin/streptomycin (Sigma, Cat # P4333). HEK 293 Gβ_1_γ_2_ stable cells were described previously (35) and were supplemented with 10% FBS, 1% penicillin/streptomycin, and 0.5 mg/ml of G418 (Invivogen, Cat # ant-gn-5). OCM-3, 92.1, and OMM1.3 cells were provided by Dr Andrew Aplin and have been previously described (19). UM001 cells were also described previously and obtained from Dr Takami Sato (57). The Mel202 cell line was from Dr Bruce Ksander. The OCM-3, 92.1, Mel202, OMM1.3, and UM001 cells were cultured in RPMI 1640 with L-Glutamine and 25 mM Hepes (Corning, Cat # 10-041-CV) supplemented with 10% FBS. All cell culture plates were obtained from Costar, Fisher, or GenClone.

### Dual luciferase assay

HEK 293 Gα_q/11_ KO cells were cultured in 12-well plates. Cells were transfected with the corresponding Gα_q_ and/or Gα_o_ construct, along with the Renilla luciferase control plasmid and either the 8x-GTIIC TEAD luciferase or the SRE luciferase reporter plasmids. The 8x-GTIIC TEAD luciferase reporter plasmid was gifted from Stefano Piccolo (Addgene plasmid # 34615). The SRE luciferase reporter plasmid was described previously (58). Lipofectamine 2000 was used for the transfections according to manufacturer's instructions. The media was changed to serum free media, with or without 1 μM YM, 2 h after transfection. After approximately 16 h, the cells were lysed in 1× passive lysis buffer (Promega, Cat # E1941) according to manufacturer's instructions. Cell lysates were plated in triplicate in an opaque white 96-well plate, and luciferase activity was detected using the Dual-Luciferase Reporter Assay System kit and the GloMax Explorer luminometer per manufacturer's instructions. 5x-SDS-PAGE sample buffer with 3.5% β-mercaptoethanol was also added separately to cell lysates and ran on a 10% SDS-PAGE gel and further immunoblotted for Gα_q_ or Gα_o_ to measure relative protein expression.

### Immunofluorescence microscopy

HEK 293 Gα_q/11_ KO cells were seeded onto coverslips in 6-well plates. For the Gα_q_-I25A experiments, pcDNA3, WT Gα_q_, or the corresponding CA Gα_q_ mutants with or without the I25A mutation were transiently transfected into the cells. For the Gα_o_ experiments, YFP-tagged Gα_q_ constructs were cotransfected into cells with 250 ng of Gα_o_ or Gα_o_ G2A. Twenty four hours after transfection, the media was changed to serum-free media with or without 1 μM YM. After approximately 16 h, the cells were fixed with 3.7% formaldehyde in PBS for 15 min. The cells were washed 3 times with PBS and blocked for 20 min in 2.5% milk in tris-buffered saline (TBS) with 1% Triton X-100. For the Gα_q_-I25A experiments, the cells were incubated with the anti-rabbit Gα_q_ antibody (Abcam) and the anti-mouse YAP antibody in 2.5% milk/TBS-Triton X-100. For the Gα_o_ experiments, the cells were incubated with the anti-rabbit Gα_o_ antibody and the anti-mouse YAP antibody in 2.5% milk/TBS-Triton X-100. The primary antibodies were incubated for 60 min. The cells were washed 5 times with 2.5% milk/TBS-Triton X-100. For the Gα_q_-I25A experiments, the cells were incubated with goat anti-rabbit Alexa Fluor 488 and goat anti-mouse Alexa Fluor 594 antibodies in 2.5% milk/TBS-Triton X-100. For the Gα_o_ experiments, the cells were incubated with goat anti-rabbit Alexa Fluor 594 and goat anti-mouse Alexa Fluor 647 antibodies in 2.5% milk/TBS-Triton X-100. The secondary antibodies were incubated for 30 min. The cells were washed 5 times in TBS/1% Triton X-100. The cells were incubated in DAPI (Thermo Fisher Scientific, Cat#D1306) diluted in warmed PBS for 5 min. The coverslips were rinsed in distilled water and mounted onto glass slides with ProLong Diamond Anti-fade Mountant (Invitrogen, Cat # P36970). The images were acquired using the Olympus IX83 microscope with a 60× oil immersion objective and an ORCA Fusion sCMOS camera (Hamamatsu) controlled by Olympus cellSens software.

### Gβ_1_γ_2_ pull-down assay

The Gβ_1_γ_2_ pull-down assay was done as previously described (35). Briefly, the HEK 293 Gβ_1_γ_2_ stable cells were seeded in 6-cm plates. For the experiments with the Gα_q_-I25A mutants, pcDNA3, WT Gα_q_, or the CA Gα_q_ mutants with or without the I25A mutation were transiently transfected into the cells. For the Gα_o_ experiments, the cells were transfected with WT Gα_q_ or the CA Gα_q_ mutants. The CA Gα_q_ mutants were cotransfected with or without 300 ng of Gα_o_ or Gα_o_ G2A. After 48 h, the cells were washed with PBS and lysed in 500 μl of lysis buffer C (20 nM Hepes pH 7.5, 100 mM NaCl, 5 mM MgCl_2_, 1 mM EDTA, and 0.7% Triton X-100, which were supplemented with the protease inhibitors 2 μg/ml leupeptin, 2 μg/ml aprotinin, and 0.1× cOmplete mini protease inhibitor cocktail). The cell lysates were incubated for 1 h on ice and were then centrifuged at 13,000 rpm (10,000*g*) to pellet the nuclei and insoluble material. Forty microliters of the lysate were reserved separately for the input fraction. The remaining lysate was added to 30 μl of Ni-NTA beads (New England BioLabs, Cat # S1423S) and rotated in an end-over-end rotator for 2 h at 4 °C. The tubes were placed in a magnetic rack, and the remaining supernatant was aspirated. The beads were washed 3 times with lysis buffer C. Fifty microliters of elution buffer (lysis buffer C with 0.25 M Imidazole) were added to elute Gβ_1_ and any bound proteins from the Ni-NTA beads. Forty microliters of the pull-down eluate were transferred to a new tube. Ten microliters of 5× SDS-PAGE sample buffer with 3.5% β-mercaptoethanol were added to the input fraction and the pull-down fraction. The input and pull-down lysates were separated on a 10% SDS-PAGE gel and protein bound was detected *via* immunoblotting. The blots were probed with the HA-tag and Myc-tag antibody (Cell Signaling Technology). The blots for the Gα_o_ experiments were also probed with Gα_o_ and GAPDH. The blots were imaged on LI-COR, and the bands were quantified on the ImageJ software (https://imagej.nih.gov/ij/). The pull-down samples in Gα_q_-I25A experiments were normalized to its respective input fraction. The pull-down samples in the Gα_o_ experiments were normalized to WT Gα_q_.

### pERK/ERK immunoblotting and analysis

Cells were lysed in SDS-PAGE sample buffer. Lysates were run on a 10% SDS-PAGE gel and transferred to a nitrocellulose membrane. The membrane was blocked in 2.5% bovine serum albumin (BSA) (Sigma Aldrich, Cat # A7906-100G) in 1× TBS supplemented with 0.05% Tween 20. The blots were incubated with both pERK (Rabbit, Cell Signaling, Cat # 9101S) and ERK (Mouse, Cell Signaling, Cat # 4696S) antibodies overnight. A duplicate immunoblot was performed and was probed with either Proteintech Gα_q_ (Rabbit, Cat #13927-1-AP) for the HEK 293 Gα_q/11_ KO cells or Cell Signaling Gα_q_ (Rabbit, Cat # 14373S) for the UM cells, along with GAPDH (Mouse, Proteintech, Cat # 60004-1-Ig) as a loading control. The ERK and pERK bands were quantified by densitometry using the ImageJ software, and the relative pERK signal was divided by ERK. The quantified signals were normalized to WT Gα_q_ (Fig. 1*D*), the individual CA Gα_q_ mutants (Fig. 4*B*), or control siRNA between the CA Gαq mutants (Fig. 6*B*) or the UM cell lines (Fig. 6*D*).

### Gβ_1/2_ siRNA transfections

Cells were plated in 6-well plates for the HEK 293 Gα_q/11_ KO cells and 12-well plates for the UM cells. For the HEK 293 Gα_q/11_ KO experiments, 30 pmol of control, Gβ_1_, Gβ_2_, or Gβ_1/2_ siRNA were transfected into cells using Lipofectamine RNAiMax (Invitrogen, Cat # 13778-150), according to manufacturer's instructions. The media was changed after 5 h. After 24 h, the corresponding WT Gα_q_, Gα_q_-QL, or Gα_q_-QP constructs were transfected into the cells using Lipofectamine 2000 (Invitrogen, Cat # 11668-019). The media was changed to serum-free media after 24 h and corresponding cells were treated with 1 μM of YM. The cells were lysed in 1× SDS-PAGE sample buffer with 0.7% β-mercaptoethanol after 16 h. For experiments with the UM cells, 15pmol of control or Gβ_1/2_ siRNA were transfected into cells using Lipofectamine RNAiMax (Invitrogen, Cat # 13778-150). The media was changed after 5 h. After 72 h, the media was changed to serum-free media, and the corresponding cells were treated with 1 μM of YM. The cells were further lysed after approximately 16 h in 1× SDS-PAGE sample buffer with 0.7% β-mercaptoethanol. The lysates were run on 10% SDS-PAGE gels and were immunoblotted for pERK (Cell Signaling, Cat # 9101S), ERK (Cell Signaling, Cat # 4696S), Gβ_1_ (Abcam, Cat # ab137635), Gβ_2_ (Abcam, Cat # ab108504), GAPDH (Proteintech, Cat # 60004-1-Ig), and Proteintech Gα_q_ (Cat #13927-1-AP) for the HEK 293 Gα_q/11_ KO cells or Cell Signaling Gα_q_ (Cat # 14373S) for the UM cells.

### Western blotting

Protein lysates were run on 10% SDS-PAGE gels and transferred to LI-COR nitrocellulose membranes (Cat # nc9680617). The membranes were blocked in either 2.5% BSA or 2.5% milk in 1× TBS/0.05% Tween 20 at room temperature for 60 min. The blots were incubated in corresponding primary antibodies in 2.5% BSA or milk in 1× TBS/0.05% Tween 20 at 4 °C overnight. The blots were washed three times in 1× TBS/0.05% Tween 20 and incubated in LI-COR anti-rabbit (Cat # 92568071) and anti-mouse (Cat # 92532212) secondary antibodies for 60 min at room temperature. The immunoblots were further washed three times in 1× TBS/0.05% Tween 20 and once with PBS. The blots were then imaged on the LI-COR Odyssey imager.

### Statistical analysis

GraphPad Prism was used to analyze the data for all of the figures. A two-way ANOVA followed by Šidák's or Tukey’s (Fig. 6, *B* and *D*) multiple comparison test were used to calculate significance. Error bars in all experiments indicate mean ± SD with significant differences indicated as ∗*p* < 0.05; ∗∗*p* < 0.01; ∗∗∗*p* < 0.005; ∗∗∗∗*p* < 0.0001.

## Data availability

All data is contained within the article.

## Supporting information

This article contains supporting information.

## Conflict of interest

The authors declare that they have no conflicts of interest with the contents of this article.
