# Supplementary material for: Disruption of the interaction between mutationally activated Gαq and Gβγ attenuates aberrant signaling
Source: J Biol Chem. 2023 Jan 7;299(2):102880. doi: 10.1016/j.jbc.2023.102880 (PMC9926304; doi:10.1016/j.jbc.2023.102880)
Supplement: Supplemental Figure S1 [file mmc1.pdf]

Supplementary Figure 1

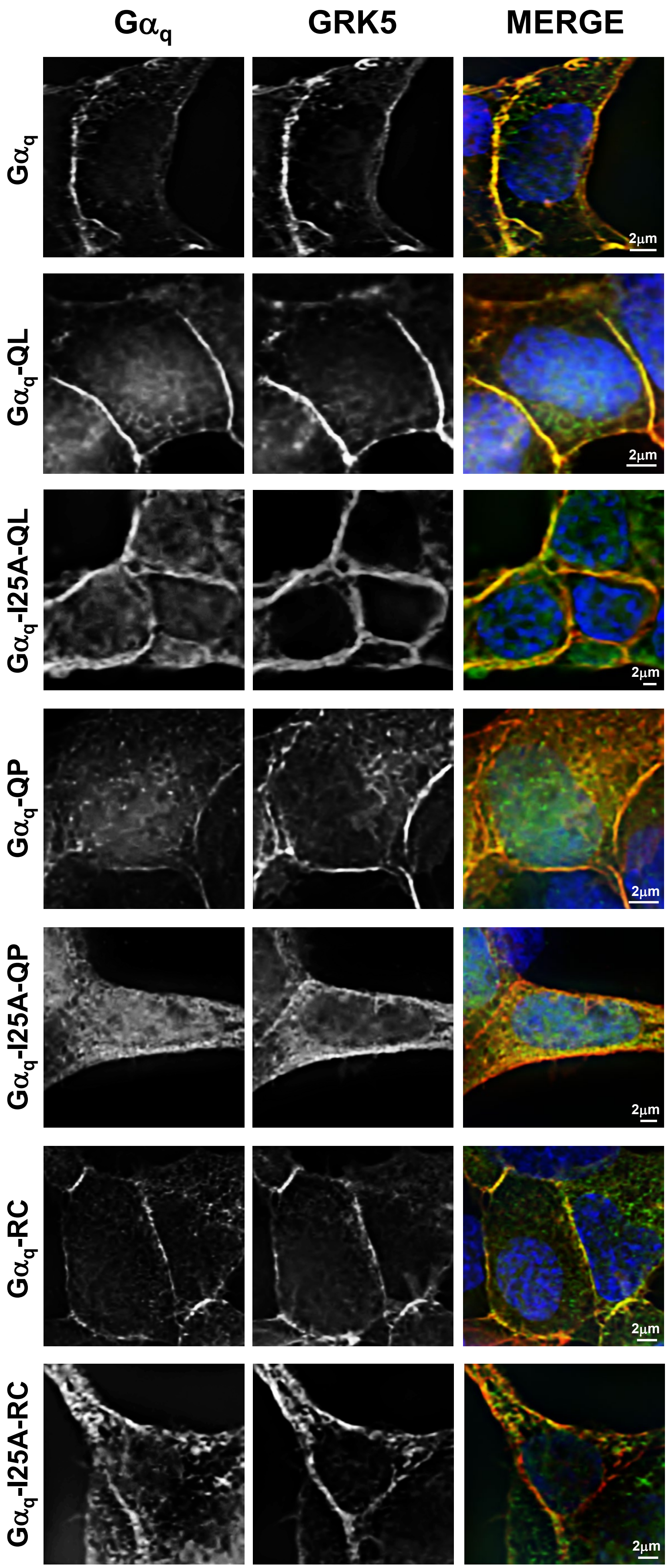

Supplementary Figure S1: **Constitutively active Gα_q and Gα_q-I25A mutants have differential cellular localization.** HEK 293 G$\alpha_{q/11}$ KO cells were seeded onto poly-L-lysine coated coverslips and transfected with a plasmid expressing WT Gα_q, Gα_q-I25A, Gα_q-QL, Gα_q-I25A-QL, Gα_q-QP, Gα_q-I25A-QP, Gα_q-RC, or Gα_q-I25A-RC, along with a GRK5 expression plasmid. GRK5 strongly localizes to the plasma membrane and is used here as a marker of the plasma membrane. Coverslips were processed for immunofluorescence microscopy to detect Gα_q, GRK5, and nuclei (DAPI) as described under Experimental Procedures, and representative images are shown (n=3). Coverslips were incubated with an anti-rabbit Gα_q antibody (Abcam) and anti-mouse GRK4-6 antibody (Sigma-Aldrich) to detect Gα_q and GRK5 expression, respectively. To remove background fluorescence, images were subjected to constrained iterative deconvolution.
